# Editorial: Computational Medicine in Visual Impairment and Its Related Disorders

**DOI:** 10.3389/fmed.2022.857485

**Published:** 2022-03-03

**Authors:** Wei Wang, Xiaoqing Gao, Yi Zhu, Erping Long

**Affiliations:** ^1^State Key Laboratory of Ophthalmology, Zhongshan Ophthalmic Center, Sun Yat-sen University, Guangzhou, China; ^2^Center for Psychological Sciences, Zhejiang University, Hangzhou, China; ^3^Department of Molecular and Cellular Pharmacology, University of Miami Miller School of Medicine, Miami, FL, United States; ^4^Division of Cancer Epidemiology and Genetics, National Cancer Institute, National Institutes of Health, Bethesda, MD, United States

**Keywords:** visual impairment, computational medicine, machine learning, data science, vision science

An estimated 254 million cases are visually impaired worldwide in 2019 (1). Visually impaired individuals often suffer from disabilities [e.g., hindered physical activities (2)] and related disorders (e.g., behavioral changes) (3), which cause a significant socio-economic burden to them. Computational medicine, fueled by advanced computational analytics and abundant datasets, has shown great potentials in the field of vision science and clinical ophthalmology. Specifically, electronic medical records and large-scale cohorts provide sufficient sample size and representative information for statistical analyses. Sophisticated algorithms (e.g., machine learning) can utilize the benefits of large-scale data to achieve automatic detection and prediction. Moreover, high-resolution imaging techniques, such as fMRI, can be empowered by computation and clinical-oriental designs (4). Given that many causes of visual impairment are preventable and treatable, these computational strategies can improve clinical outcomes and contribute to a better understanding of vision science.

A Research Topic was hosted by the section of Ophthalmology of Frontiers in Medicine, which covers recent advances of computational medicine in visual impairment. In this Research Topic, we asked three general questions: (1) How could machine learning algorithms transform the management of visually impaired patients? (2) Do data-driven medical research show clear values in guiding clinical practice? (3) Can clinical-oriental research provide novel insights into vision science?

The first question about machine learning received response from four contributed studies. Jiang et al. who designed a deep-learning-based lens partition strategy in slit-lamp images, a routine procedure in ophthalmology. Focusing on infantile cataracts (the major cause of infant blindness worldwide), the designed strategy achieved improved generalizability in multi-center datasets, which could enable timely intervention and improved outcomes. Mishra et al. developed a novel deep-learning-based framework, named VTG-Net (vessel topology graph network), to classify retinal artery/vein (A/V) by fundus images. Compared with state-of-the-art methods, VTG-Net presented an improved performance in both public and private datasets, which can potentially assist the diagnosis of diabetic retinopathy (the most common cause of vision loss for diabetes patients). Wang et al. developed a semi-automated deep-learning system to detect diabetic retinopathy by fundus images with high accuracy, sensitivity, and specificity. Notably, their semi-automated approach can save 75.6% time and 90.1% economic cost compared with fully human grading, which represents an optimized balance among safety, time, and economic cost. Moreover, Yang et al. developed a machine-learning-based classifier to detect diabetic retinopathy by non-ocular metrics (e.g., systolic blood pressure and body mass index). Their best-performance model (XGBoost) achieved area under the receiver operating characteristic curve (AUC) of 0.816 based on top-10 non-ocular metrics ranked by feature importance. These studies leveraged the machine-learning algorithms to provide valuable assistance and paradigm shift in clinical practice in different ophthalmic contexts.

The second question about medical research was responded by Lin et al.. In this study, they assessed the benefits of health examination centers in China, a commonly established public health system to provide screening and early detection at a large population scale. In the case of refractive error, they included a total of 5,284 participants and performed cross-sectional analyses. Their results not only showed a high prevalence of uncorrected refractive error in urban China area, but also highlighted the efficiency and merit of health examination centers in refractive error service.

The third question about visual science was responded by Feng et al.. They focused on individuals with bilateral congenital cataracts, a unique scenario to explore the role of early visual experience in shaping the human cortex. Fueled by the high-resolution structural and resting-state fMRI technology, they observed structural and functional cortical changes among adults who experienced visual deprivation early in life. Their findings suggest that even a brief early visual deprivation (a few months) is sufficient to induce a long-lasting reorganization of the human cortex.

To conclude, the articles published in this Research Topic can be representatives in applying computational medicine to both medical research and clinical settings. We hope that this issue can increase the awareness of readers from the interdisciplinary contexts of medicine and computer science.

## Author Contributions

WW, XG, YZ, and EL drafted the manuscript. All authors discussed and approved the final manuscript.

## Conflict of Interest

The authors declare that the research was conducted in the absence of any commercial or financial relationships that could be construed as a potential conflict of interest.

## Publisher's Note

All claims expressed in this article are solely those of the authors and do not necessarily represent those of their affiliated organizations, or those of the publisher, the editors and the reviewers. Any product that may be evaluated in this article, or claim that may be made by its manufacturer, is not guaranteed or endorsed by the publisher.
